# Palm Tocotrienol-Rich Fraction Improves Vascular Proatherosclerotic Changes in Hyperhomocysteinemic Rats

**DOI:** 10.1155/2013/976967

**Published:** 2013-03-20

**Authors:** Ku-Zaifah Norsidah, Ahmad Yusof Asmadi, Ayob Azizi, Othman Faizah, Yusof Kamisah

**Affiliations:** ^1^Department of Basic Medical Sciences, Kulliyyah of Medicine, International Islamic University of Malaysia, 25200 Kuantan, Pahang, Malaysia; ^2^Faculty of Traditional and Complementary Medicine, Cyberjaya University College of Medical Sciences, 63000 Cyberjaya, Selangor, Malaysia; ^3^Quéstra Clinical Research Sdn Bhd, 10350 Penang, Malaysia; ^4^Department of Anatomy, Faculty of Medicine, UKMMC, Universiti Kebangsaan Malaysia, 50300 Kuala Lumpur, Malaysia; ^5^Department of Pharmacology, Faculty of Medicine, UKMMC, Universiti Kebangsaan Malaysia, 50300 Kuala Lumpur, Malaysia

## Abstract

This study investigated the effects of palm tocotrienol-rich fraction (TRF) on aortic proatherosclerotic changes in rats fed with a high methionine diet. Forty-two male Wistar rats were divided into six groups. The first group was the control (fed with a basal diet). Another five groups were fed with 1% methionine diet for 10 weeks. From week 6 onward, folate (8 mg/kg diet) or palm TRF (30, 60, and 150 mg/kg diets) was added into the diet of the last four rat groups, respectively. The high methionine diet raised the plasma total homocysteine and aortic lipid peroxidation, which were reduced by the palm TRF and folate supplementations. Plasma nitric oxide was reduced in the high methionine group compared to the control (3.72 ± 0.57 versus 6.65 ± 0.53 **μ**mol/L, *P* < 0.05), which reduction was reversed by the palm TRF (60 and 150 mg/kg) and folate supplementations. The increased aortic vascular cell adhesion molecule-1 expression in the methionine group (2.58 ± 0.29) was significantly reduced by the folate (1.38 ± 0.18) and palm TRF at 150 mg/kg (1.19 ± 0.23). Palm TRF was comparable to folate in reducing high methionine diet-induced plasma hyperhomocysteinemia, aortic oxidative stress, and inflammatory changes in rats.

## 1. Introduction

Hyperhomocysteinemia is regarded as one of the important risk factors for cardiovascular diseases such as hypertension [1, 2], the most common cause of increased morbidity and mortality in many countries [3, 4]. Many studies have shown that hyperhomocysteinemia can be induced by feeding experimental animals with a high methionine diet [5, 6]. Hyperhomocysteinemia enhances production of a reactive oxygen species (ROS), leading to increased oxidative stress [7] which then reduces nitric oxide (NO) production [8]. It has been reported to impair vascular endothelial dysfunction [9, 10] and promote early changes of atherosclerosis [11].

Atherosclerosis is a chronic inflammatory process. The earliest changes involve recruitment of monocytes, which later differentiate to macrophages in subintimal layers. The recruitment and accumulation of these macrophages require the presence of adhesion molecules such as vascular cell adhesion molecules (VCAM-1) which is important for binding and adhesion of the monocytes in the blood stream [12, 13]. Previous studies have shown that raised homocysteine level lead to increased monocyte adhesion to the vessel wall [14, 15].

Many reports have been published regarding the role of antioxidants in cardiovascular diseases. Studies that investigated the effects of vitamin E on cardiovascular diseases were mainly focused on *α*-tocopherol. Tocotrienol, another type of vitamin E has shown a promising beneficial effect on cardiovascular system in humans [16]. Several studies have demonstrated that it possesses antioxidant [17, 18], anti-inflammatory [19], and hypocholesterolemic [20] properties. We have previously shown that palm tocotrienol-rich fraction (TRF), a vitamin E extract from palm oil which contained both tocopherols and tocotrienols, reduced plasma homocysteine level and heart oxidative stress in rats fed with a high methionine diet [21].

Based on the previously mentioned reports, the objectives of this study were to determine the effects of palm TRF on hyperhomocysteinemia and vascular parameters in rats fed a high methionine diet. The effects of palm TRF on the parameters were also compared to folate, a standard intervention for hyperhomocysteinemia.

## 2. Materials and Methods

### 2.1. Animals and Chemicals

Forty-two male Wistar rats (180–200 gram) were obtained from the Laboratory Animal Resource Unit of Universiti Kebangsaan Malaysia. They were kept in polyethylene cages in a well-ventilated room at room temperature (28°C). Food and water were provided *ad libitum* based on their experimental groups. All chemicals and enzymes were purchased from Sigma-Aldrich (St. Louis, MO, USA), unless otherwise stated. The palm TRF used in this study was prepared by the Malaysian Palm Oil Board according to Gapor et al. [22], comprising 21%  *α*-tocopherol, 17%  *α*-tocotrienol, 4%  *γ*-tocopherol, 33%  *γ*-tocotrienol, and 24%  *δ*-tocotrienol. The basal diet contained about 25.11 mg/kg total vitamin E, and its composition was as follows: *α*-tocopherol acetate, 40.62%, *α*-tocopherol 21.62%, *α*-tocotrienol, 10.71%, *γ*-tocopherol, 3.46%, *γ*-tocotrienol, 18.08%, and *δ*-tocotrienol, 5.49%. It also contained 4.1 g/kg methionine and 2.4 mg folate per tonne matric food.

### 2.2. Preparation of Experimental Diet

A high methionine diet (1% methionine) [23] was prepared by mixing 990 g of basal diet (Gold Coin Feedmills Malaysia Sdn Bhd) with 10 g methionine. Arabic gum (2%) solution was added to stick them together. For the palm TRF- or folate-supplemented diets, palm TRF at 30, 60, or 150 mg/kg diets [24] or folate (8 mg/kg diet) [25] was added. The diet was mixed for 30 minutes. Using a 10-cc syringe, it was remolded and left to dry overnight. The diets were then kept at −20°C before use.

### 2.3. Experimental Design

After one week of acclimatization, the rats were randomly divided equally into six groups (*n* = 7). The first group, the control, was fed basal rat chow throughout the ten-week study period. The second group was given a high methionine diet only. The other four groups were given the high methionine diet (from weeks 1 to 5) followed by supplementation with folate (M + Folate, 8 mg/kg) or palm TRF at 30 (M + TRF30), 60 (M + TRF60), or 150 (M + TRF150) mg/kg diets from the sixth week onwards. At the end of the treatment period, systolic blood pressure levels were measured using a noninvasive tail cuff method (Physiograph, Dess, USA). The rats were then deprived of food overnight and sacrificed. The aorta was removed and washed with ice-cold buffered saline. The abdominal part of the aorta was kept at −71°C until being used for biochemical determinations, while the thoracic part was processed for histological examination. The experimental procedure and humane animal handling were conducted in accordance with the national guidelines for the care and use of laboratory animal, and were approved by the Universiti Kebangsaan Malaysia Animal Ethics Committee. Rat food intake was recorded throughout the experiment duration and reported as total food intake.

### 2.4. Determination of Biochemical Parameters

Plasma total homocysteine level was determined at weeks 0, 5 and 10. The levels were quantified by the means of commercial diagnostic kit (Abbot Laboratories, IL, US) that utilized the fluorescence polarization immunoassay technique and expressed as *μ*mol/L.

The abdominal aorta was used to measure the lipid peroxidation content [26, 27], expressed as TBARS (pmol MDA/mg protein). Plasma nitric oxide (NO_*x*_) at weeks 0 and 10 was measured using Griess reagent.

### 2.5. Immunohistochemical Staining

The thoracic aorta was immersion-fixed in 10% buffered formalin overnight and then embedded longitudinally in paraffin. Sequential 5 *μ*m paraffin-embedded sections were deparaffinized and incubated with H_2_O_2_ for 5 minutes to quench the endogenous peroxide. The sections were immunostained with primary antibody of rabbit monoclonal antibody (1 : 100) against rat VCAM-1 (Santa Cruz Biotechnology, Inc., Santa Cruz, CA) for an hour at room temperature. After washing with trizma base solution (TBS), an anti-rabbit polymerized horseradish peroxidase labeled secondary antibody (DakoCytomation) was added, and the sections were incubated for 30 minutes at room temperature. Sections were then washed, incubated in liquid diaminobenzedine for 10 minutes, washed and counterstained with hematoxylin, cleared and mounted. Slides containing human tonsil were used as positive and negative controls. Positively expressing vascular cell adhesion molecule-1 was indicated by brown streaks in the intimal layer of the aorta. The immunohistochemical staining slides were manually assessed quantitatively by two experienced operators who were blinded to the study protocol, based on the following grading under ×400 magnification: 0 = no staining, 1 = staining less than 50% intimal surface, 2 = staining more than 50% intimal surface, 3 = complete surface staining but thin, 4 = complete and thick staining of intimal surface.

### 2.6. Histomorphometric Study

Verhoeff-stained and differentiated in 2% ferric chloride cross sections from thoracic aorta were checked microscopically until the nuclei and elastic fibers were stained black and washed again in water, followed by immersion in 95% alcohol to remove excess iodine. It was later counterstained in van Gieson for 30 seconds. The structure of the sections was observed under light microscope (Eclipse 80i, Nikon Corporation, Tokyo, Japan), and the images were captured using a camera (Qimaging MicroPublisher 5.0, Surrey, Bc, Canada). The thickness of intima and media layer at 0°, 90°, 180°, and 270° of each section were quantitatively analyzed by using Image Pro Plus 5.0 (Media Cybernetics, Inc., Silver Spring, MD, USA) under ×200 magnification. The average readings of intima-media thickness and intima : media ratio were calculated.

### 2.7. Statistical Analysis

Statistical analyses were performed using Statistical Product for Social Science (SPSS) 11.5. Data are expressed as mean ± SEM. Kolmogorov Smirnov test was used to determine the normality of data distribution. As the data were normally distributed, analysis of variance (ANOVA) followed with post hoc Tukey test was used. The correlations between parameters were analyzed with Pearson correlation test. Values of *P* < 0.05 were considered statistically significant. 

## 3. Results

### 3.1. Food Intake

**Figure 1:**
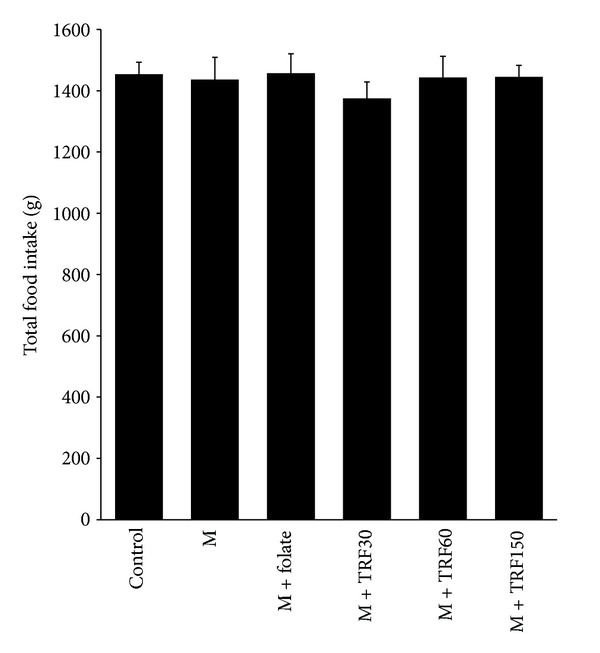
Total food intake in rats fed with a high methionine diet (M) (weeks 1–10) and supplemented with palm tocotrienol-rich fraction (TRF) at three different doses (30, 60, and 150 mg/kg diets) or folate (8 mg/kg diet) for 5 weeks (weeks 6–10). Bars represent means ± SEM (*n* = 7). No significant difference was seen between groups.

Mean total food intake in all experimental groups is shown in Figure 1. The mean total intake was about 1450 g for the duration of 10 weeks. It was similar in all groups, and no significant difference was observed amongst the groups.

### 3.2. Plasma Total Homocysteine

**Figure 2:**
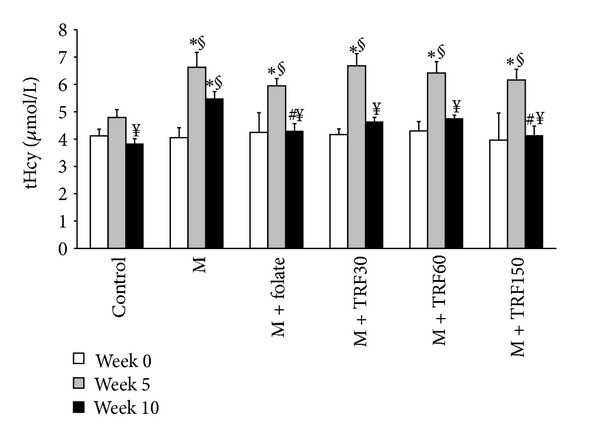
Total plasma homocysteine level at weeks 0, 5, and 10 in rats fed high methionine diet (M) (week 0–10) and supplemented with either folate (8 mg/kg diet) or palm tocotrienol-rich fraction (TRF) (30, 60, or 150 mg/kg diets) for 5 weeks (weeks 6–10). The result is expressed as means ± SEM (*n* = 7). *Significantly different from the control at the same duration of treatment (*P* < 0.05), ^#^significantly different from the methionine group at week 10 (*P* < 0.05), ^§^significantly different from week 0, and ^¥^significantly different from week 5, respectively (*P* < 0.05).

Plasma total homocysteine levels (Figure 2) at week 5 were significantly raised in all rat groups that were fed 1% methionine diet compared to the control group, and no difference was seen amongst the groups fed methionine diet. At week 10, the plasma total homocysteine levels in all supplemented groups (folate and palm TRF) were significantly lower compared to their respective groups at week 5. However, only reductions at week 10 in groups supplemented with folate and palm TRF at 150 mg kg diet were significantly lower than the high methionine group (at week 10). In the control group, a lower plasma total homocysteine was also observed. However, there was no significant difference seen in the homocysteine levels amongst the supplemented and control groups at week 10. No significant difference was observed in all groups at week 0.

### 3.3. Aortic TBARS Content

**Figure 3:**
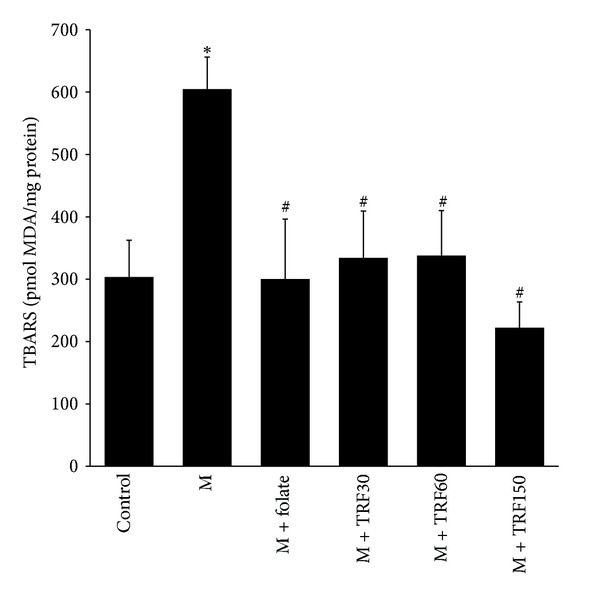
The thiobarbituric acid reactive substance (TBARS) content (pmol MDA/mg protein) in aorta of rat fed either basal (control), high methionine (M, 1%) (week 0–10), methionine + folate (8 mg/kg diet) or palm tocotrienol rich fraction (TRF) (30, 60 or 150 mg/kg) diets (week 6–10). Bars represent means ± SEM (*n* = 7). *Significantly different from the control (*P* < 0.05), ^#^Significantly different from the M group (*P* < 0.05).

There was almost a 100% increase in the aortic TBARS content following the high methionine diet compared to the control (604.0 ± 51.0 versus 300.3 ± 58.7 pmol MDA/mg protein) (Figure 3). In rats that received folate (300.3 ± 96.2 pmol MDA/mg protein) and palm TRF supplementation (30 mg/kg, 334.0 ± 75.4, 60 mg/kg, 337.8 ± 72.3, and 150 mg/kg, 222.3 ± 41.2 pmol MDA/mg protein) for 5 weeks in addition to the high methionine diet, the TBARS contents were significantly lower compared to the methionine group. No difference amongst the palm TRF- and folate-supplemented groups was observed.

### 3.4. Plasma Nitric Oxide

**Figure 4:**
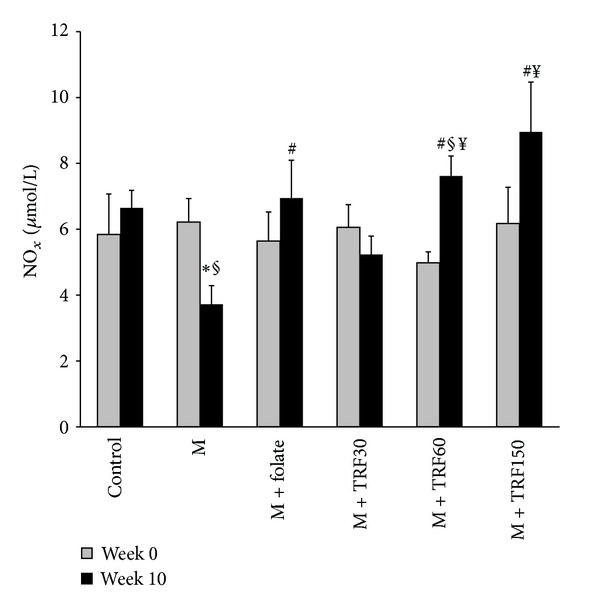
Plasma nitric oxide in rats given high methionine (M) (1%) diet for 10 weeks and supplemented with palm tocotrienol rich fraction (TRF, 30, 60 and 150 mg/kg diet from weeks 6 to 10) or folate (8 mg/kg diet). Bars represent means ± SEM (*n* = 7). *Significantly different from the control (*P* < 0.05), ^#^significantly different from the M group (*P* < 0.05), ^§^significantly different from week 0 respectively and ^¥^significantly different from the TRF30-treated group (*P* < 0.05).

Intake of the high methionine diet for 10 weeks significantly decreased plasma nitric oxide compared to the control (3.72 ± 0.57 versus 6.65 ± 0.53 *μ*mol/L, *P* < 0.05) and its own group at week 0 (Figure 4). Groups supplemented with folate and palm TRF at 60 and 150 mg/kg diets increased the plasma level significantly compared to the methionine group. Both groups supplemented with 60 and 150 mg/kg palm TRF had significantly higher plasma nitric oxide than the group supplemented palm TRF at low dose (30 mg/kg) (7.62 ± 0.61 and 8.96 ± 1.51 versus 5.23 ± 0.56 *μ*mol/L, *P* < 0.05). The plasma levels in these two groups were not significantly different. No significant difference was seen amongst the groups at week 0.

### 3.5. Aortic VCAM-1 Expression

**Figure 5:**
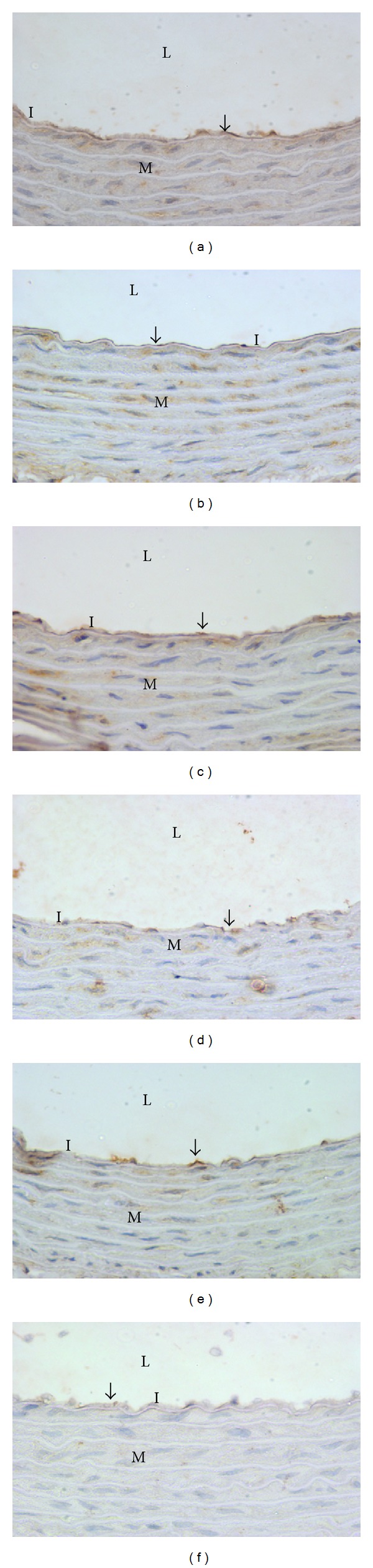
Intimal layer of aorta positively expressing vascular cell adhesion molecule-1 (VCAM-1, brown streaks indicated by arrows) (×400 magnification) in rats fed basal (control, (a)), high methionine (1%, (b)) (weeks 0–10) diets and supplemented with folate (8 mg/kg, (c)) or palm tocotrienol-rich fraction (TRF) at 30 (d), 60 (e), or 150 (f) mg/kg for 5 weeks (weeks 6–10), in addition to high methionine diet. L: lumen, I: intima, and M: media.

The representatives of the aortic intimal layer positively expressing VCAM-1 (indicated by brown stained streak) from each group are demonstrated in Figure 5. The positive-stained area was significantly observed in the methionine group. While in the folate- and palm TRF-supplemented groups, the area were less intensified. 

**Figure 6:**
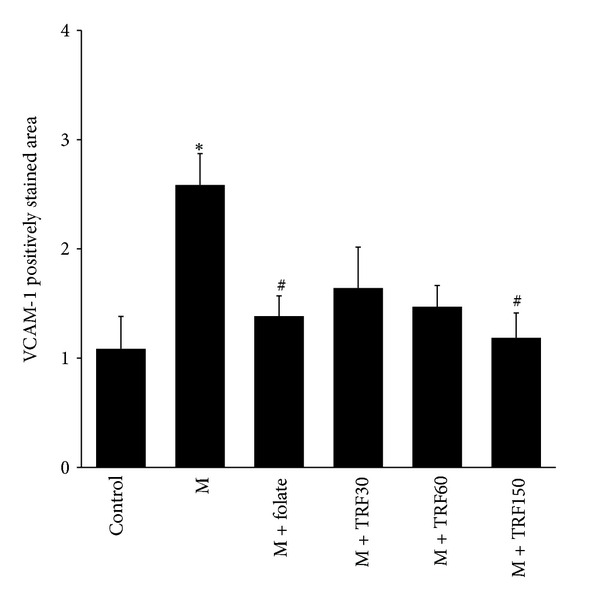
The effects of folate (F; 8 mg/kg diet) and palm tocotrienol-rich fraction (TRF; 30, 60, and 150 mg/kg diets) for 5 weeks (From weeks 6 to 10) on aortic vascular cell adhesion molecule 1 (VCAM-1) expression in rats fed high methionine diet (M) (From weeks 0 to 10). Bars represent means ± SEM (*n* = 7). *Significantly different from the control (*P* < 0.05) and ^#^significantly different from the methionine group (*P* < 0.05).

The high methionine diet (2.58 ± 0.29 versus 1.09 ± 0.29, *P* < 0.05) caused a significant increase in the area of aortic intimal layer that was positively expressing VCAM-1 (Figure 6). The supplementations of folate (1.38 ± 0.18) and palm TRF at 150 mg/kg diet (1.19 ± 0.23) significantly reduced the expression of VCAM-1. Palm TRF at 30 or 60 mg/kg however, did not influence the expression of VCAM-1 significantly. 

### 3.6. Aortic Histomorphometric Study

**Figure 7:**
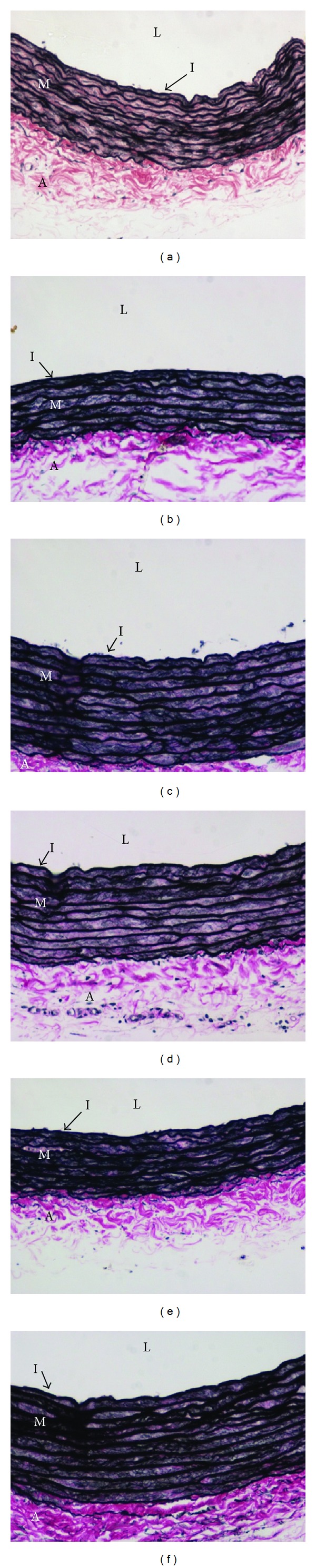
Verhoeff van Gieson staining of the aorta (×200 magnification) in rats fed basal (control, (a)), high methionine (weeks 0–10, (b)) diets and supplemented with folate (8 mg/kg diet, (c)) or palm tocotrienol-rich fraction (TRF) at 30 (d), 60 (e), or 150 (f) mg/kg diets) for 5 weeks (week 6–10) in addition to high methionine diet. L: lumen, I: intima, M: media, and A: adventitia.

The Verhoeff van Gieson-stained cross-sections showing the intima and media of the aorta from each group were shown in Figure 7. The aortic intima-media thickness and intima : media ratio were not influenced by the dietary high methionine. Supplementations of neither folate nor palm TRF at all doses significantly affected these parameters (Table 1). 

### 3.7. Systolic Blood Pressure

**Figure 8:**
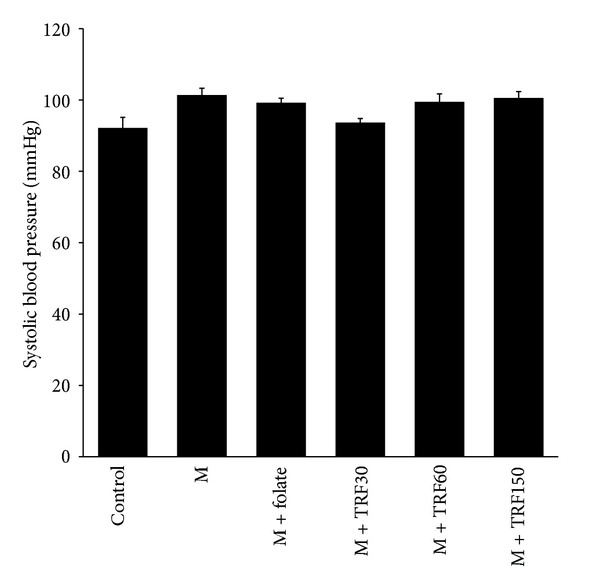
Systolic blood pressure in rats given high methionine (M) (1%) diet (From weeks 1 to 10) and supplemented with palm tocotrienol-rich fraction (TRF; 30, 60, and 150 mg/kg diets from weeks 6 to 10) or folate (8 mg/kg diet). Bars represent means ± SEM (*n* = 7). No significant different was seen amongst groups.

The high methionine diet for 10 weeks did not significantly influence the systolic blood pressure in rats (101.37 ± 1.92 versus 92.15 ± 2.98 mmHg). Supplementations of palm TRF (30 mg/kg, 93.65 ± 1.15, 60 mg/kg, 99.48 ± 2.22, and 150 mg/kg, 100.58 ± 1.79 mmHg) and folate (99.23 ± 1.24 mmHg) also had no significant effect on this parameter (Figure 8).

### 3.8. Relationships between Parameters

Plasma total homocysteine level was positively correlated to the aortic VCAM-1 (*r* = 0.339, *P* < 0.05) and TBARS (*r* = 0.369, *P* < 0.05). Aortic TBARS was also found to be positively correlated to the aortic VCAM-1 (*r* = 0.364, *P* < 0.05). However, it demonstrated a negative correlation to plasma nitric oxide (*r* = −0.321, *P* < 0.05) (Table 2).

## 4. Discussion

A high methionine diet model is an established experimental model for the induction of hyperhomocysteinemia. In the present study, the plasma total homocysteine level was increased following ingestion of 1% methionine diet, which confirms previous data demonstrating that hyperhomocysteinemia could be induced by a high methionine diet [5, 6]. No difference seen in the mean total food intake in all groups suggests that the intake of methionine in all groups except in the control group was similar. The average daily food intake was about 20 g per rat, which means that in the methionine fed groups, each rat consumed approximately 250–280 mg of methionine daily. While in the control group, the methionine consumption was only 80–85 mg. The basal methionine content in the rat chow did not significantly increase the plasma homocysteine, as seen in the present study. 

In the body, methionine from the diet is converted to S-adenosyl-methionine and S-adenosyl-homocysteine and finally to homocysteine. The homocysteine is then remethylated to methionine by the action of methionine synthase, or it undergoes transsulfuration pathway through which it is converted into cysteine by cystathionine-*β*-synthase [28]. Vitamin B12 and folate act as important coenzymes for homocysteine remethylation in the folate cycle, which results in a reduced level of homocysteine [29]. This explains the reduction of the plasma total homocysteine level seen in the rats that were supplemented with folate in the present study. Folate supplementation has been reported by other researchers to significantly reduce plasma homocysteine level in experimental animals [14, 30] and humans [31, 32]. 

Recent epidemiological studies reported that folic acid supplementation to lower homocysteine levels had no significant beneficial effects on vascular outcomes [33, 34]. However, there were other epidemiological data that had shown otherwise. The epidemiological review had found that homocysteine lowering effect of B vitamins supplementation showed a significant protective effect on stroke [35] and in preventing vascular events as well as cardiovascular diseases mortality in high risk individuals [36]. 

Reductions in the plasma total homocysteine level in all palm TRF-supplemented groups, were comparable to that observed in the folate-supplemented group and its effect was not dose dependent. It was shown that *α*-tocopherol supplementation for two months had failed to reduce plasma homocysteine in highly trained athletes [37]. Similarly, a combination of antioxidants which consisted of *α*-tocopherol, *β*-carotene, vitamin C, troxerutin, and selenium did not influence the plasma homocysteine level in patients with hyperhomocysteinemia [38]. The discrepancy in the effect of both antioxidants, tocopherol and tocotrienol, on the blood parameter is not well understood. It could be that palm TRF which contained more than 70% tocotrienol reduced plasma total homocysteine by affecting the enzymes and cofactors involved in the homocysteine metabolism. This probable effect might be demonstrated by a different mechanism, not via its antioxidant effect. This postulation warrants further investigation. The reduction seen in the control group at week 10 compared to the level at week 5 was also not understood. However, the level was comparable to the levels in the supplemented groups at week 10.

Hyperhomocysteinemia induced by high methionine diet increased vascular oxidative stress, as shown by increased aortic TBARS. The increase in the plasma total homocysteine level was positively correlated with the increase in oxidative stress. A similar finding was also reported by Yu et al. [39]. Homocysteine increased the reactive oxygen species generations, namely, superoxide and peroxynitrite mediated by NADPH oxidase in vascular cells, leading to endothelial dysfunction [40]. The supplementations of folate and all doses of palm TRF prevented the increase in aortic TBARS induced by high methionine diet, at similar efficacy. The protective effect of palm TRF to reduce oxidative stress has been previously reported [17, 18]. It was also reported to prevent the increase in heart oxidative stress in hyperhomocysteinemic rats [21]. The ability of *α*-tocopherol to inhibit the increased reactive oxygen species generation induced by homocysteine was also seen in vascular smooth muscle cells [41]. Folate was also reported to possess a good antioxidant property [42, 43]. 

In rats that were fed high methionine diet, a significant reduction in the plasma nitric oxide was observed. Homocysteine was directly shown to inhibit nitric oxide synthase activity which later resulted in decreased nitric oxide level [44]. A significant association between the homocysteine and nitric oxide levels was not shown in our study, even though He et al. [45] had previously reported their significant negative correlation in human subjects. Nitric oxide is important to blood vessels due to its vasorelaxant effect. Diminished nitric oxide bioavailability is accompanied by an increase in oxidative stress [46, 47], and the significant negative correlation between these two parameters was demonstrated in this study. The palm TRF supplementations at 60 and 150 mg/kg diets and folate managed to significantly prevent the loss of plasma nitric oxide. 

In our study, the high methionine diet-induced hyperhomocysteinemia increased the expression of aortic VCAM-1, an inflammatory biomarker, and was in agreement with the study of Li et al. [14]. They had shown that high methionine diet raised plasma homocysteine level and subsequently augmented the expression of VCAM-1 in rat thoracic aorta. Indeed, VCAM-1 plays a more important role than intracellular adhesion molecule (ICAM-1) in the early progression of atherosclerosis [48]. Its significant association with hyperhomocysteinemia was confirmed in the present study which suggests that in hyperhomocysteinemia, the formation of proatherosclerotic marker in the blood vessels is a part of the pathological changes. It has a crucial role in leukocytes recruitment into the inflamed sites in the vascular tissues [49], by mediating the adhesion of leukocytes to the surface of endothelial cells, thus promoting smooth muscle cell migration through the endothelium into the intima during atherogenesis [50]. The VCAM-1 increased expression was shown to be related to increased oxidative stress in fructose-fed rats [51] and their positive correlation was demonstrated in our study. However at this stage, hyperhomocysteinemia did not affect aortic histomorphometric changes, calculated as intima-media thickness and intima : media ratio, as well as no effect on systolic blood pressure. 

Clinically, indirect measurement of intimal thickness of arteries, like carotid and femoral, can be used to study disease progression and effects of treatment in cardiovascular diseases. Previous reported studies had exhibited no relationship between hyperhomocysteinemia and carotid intima-media thickness [52, 53]. However, Sprague-Dawley rats fed a 1% methionine diet for a month showed a significant increase in the intima : media ratio which was associated with a four-fold increase in homocysteine level [54], but we only found an approximately two-fold increment in our study. 

A study carried out by Robin et al. [55] showed that the systolic blood pressure was elevated in normotensive rats supplemented with 1.45% methionine after 7 weeks, a dose higher than the present study. Their study also confirmed that the positive association between plasma homocysteine and blood pressure was unlikely to be causal. The increased plasma homocysteine seen in the hypertensive rats was accompanied with a significant decrease in blood pressure instead. It can be postulated that in our study the early atherosclerotic changes induced by hyperhomocysteinemia had already taken place in the blood vessels before the manifestation of hypertension.

Similar to the high methionine group, both palm TRF and folate did not significantly affect the vascular histomorphometry and systolic blood pressure. However, palm TRF at the highest dose (150 mg/kg diet) and folate suppressed vascular proatherosclerotic changes in the hyperhomocysteinemic rats. This finding was indicated by normalized expression of VCAM-1. Tocopherols and tocotrienols were demonstrated to inhibit inflammatory biomarker *in vitro* which resulted in a reduced endothelial invasiveness and leukocyte attachment [56, 57]. For tocotrienols, the suppression was the highest in cells treated with *δ*-tocotrienol (77%) followed by *β*-tocotrienol (63%), *γ*-tocotrienol (54%), *α*-tocotrienol (38%), and the least with *α*-tocopherol (29%). The difference in efficacy of the vitamin E isomers could be related to the higher antioxidant function of the tocotrienol compared to the tocopherol, due to its higher mobility in cell membrane and better recycling of its chromanoxyl radical [57]. Similarly, the suppressive effect by the tocopherols was the highest with *δ*-tocopherol, followed by *γ*-tocopherol, and the least being *α*-tocopherol [56]. Collectively, it can be concluded that the inhibitory effect of palm TRF on VCAM-1 expression is primarily mediated by its tocotrienol content. As for folate, it has been shown to reduce VCAM-1 expression in hyperhomocysteinemic rat aorta [14]. This study has confirmed the anti-inflammatory property of both folate and palm TRF, in addition to their antioxidant effect. 

## 5. Conclusions

This study demonstrated that a high methionine diet-induced hyperhomocysteinemia was associated with increased aortic oxidative stress and inflammatory changes in male adult rats. Dietary supplementation of palm TRF particularly at the highest dose (150 mg/kg) was shown to be effective in reducing the plasma total homocysteine, aortic oxidative stress, and inflammatory changes, comparable to that of folate. 

## Figures and Tables

**Table 1:** The aortic intima and media ratio in rats fed 1% methionine diet (M) (week 0–10), and supplemented with folate (8 mg/kg diet) or palm tocotrienol rich fraction (TRF) (30, 60 or 150 mg/kg diet) for 5 weeks (week 6–10).

Groups	Intima-media thickness (*μ*m)	Intima : media ratio
Control	95.78 ± 7.19	0.0163 ± 0.0017
Methionine (M)	89.02 ± 7.98	0.0160 ± 0.0011
M + Folate	92.11 ± 8.33	0.0157 ± 0.0016
M + TRF30	98.05 ± 8.26	0.0170 ± 0.0020
M + TRF60	84.41 ± 7.95	0.0154 ± 0.0023
M + TRF150	82.74 ± 9.88	0.0146 ± 0.0018

Values are means ± SEM (*n* = 7). No significant difference amongst groups.

**Table 2:** Correlation (*r*) between parameters in rats fed high methionine diet.

	Aortic VCAM-1	Plasma NO_*x*_	Aortic TBARS
Plasma total homocysteine	0.339*	−0.049	0.369*
Aortic TBARS	0.364*	−0.321*	
Plasma NO_*x*_	−0.300		

*Significant correlation (*P* < 0.05).
